# Novel Arenavirus, Zambia

**DOI:** 10.3201/eid1710.10452

**Published:** 2011-10

**Authors:** Akihiro Ishii, Yuka Thomas, Ladslav Moonga, Ichiro Nakamura, Aiko Ohnuma, Bernard Hang’ombe, Ayato Takada, Aaron Mweene, Hirofumi Sawa

**Affiliations:** Hokkaido University, Sapporo, Japan (A. Ishii, Y. Thomas, I. Nakamura, A. Ohnuma, A. Takada, H. Sawa);; University of Zambia, Lusaka, Zambia (A. Ishii, Y. Thomas, L. Moonga, I. Nakamura, B. Hang’ombe, A. Takada, A. Mweene, H. Sawa)

**Keywords:** Arenavirus, arenavirus infections, Mastomys natalensis, Zambia, Republic of Zambia, phylogeny, Old World arenaviruses, viruses, hemorrhagic fevers, viral, dispatch

## Abstract

To investigate arenavirus in Zambia, we characterized virus from the kidneys of 5 arenavirus RNA–positive rodents (*Mastomys natalensis*) among 263 captured. Full-genome sequences of the viruses suggested that they were new strains similar to Lassa virus–related arenaviruses. Analyzing samples from additional rodents and other species can elucidate epizootiologic aspects of arenaviruses.

Arenavirus, a bisegmented ambisense single-stranded RNA virus, is 1 of the viral pathogens responsible for hemorrhagic fever in Africa and South America. Until 2007, Lassa virus was the only known arenavirus to cause hemorrhagic fever in Africa. However, during September–October 2008, 5 hemorrhagic fever cases caused by a novel arenavirus named Lujo virus occurred in South Africa (*1*). The initial case occurred in Zambia; the patient was transported to South Africa for treatment, where the virus spread to 4 other persons. Four patients died; the source of infection in the index patient was not determined.

The natural reservoir of arenavirus in Africa is rodents of the family Muridae, especially *Mastomys natalensis,* and nonpathogenic arenaviruses have been found in areas surrounding Zambia (*2**–**5*). To further the epizootiologic understanding of arenaviruses, we investigated their prevalence and genetic background among *M. natalensis* rodents in Zambia during May 22–August 28, 2009.

## The Study

We conducted the study with permission from the Zambia Wildlife Authority. Sherman traps (H.B. Sherman, Inc., Tallahassee, FL, USA) were set up on dry, arable lands or scrublands surrounding the cities of Lusaka (15°26′30.85′′S, 28°26′51.09′′E), Namwala (15°43′01.37′′S, 26°42′33.41′′E), and Mfuwe (13°6′47.92′′S, 31°48′17.24′′E). We captured 57, 48, and 158 rodents in each of these cities, respectively. Rodents were euthanized with diethyl ether, and kidney tissues were harvested and stored at −80°C.

For species identification of the rodents, DNA was extracted by using the DNeasy Blood and Tissue Kit (QIAGEN, Chuo-ku, Tokyo). *Mastomys* spp. were identified by mitochondrial cytochrome b gene (*cytb*) (*6*). Two primers, mCytb-F (5′-ACCCACTGTTTAAAATTATTAACCACTC-3′) and mCytb-R (5′-CTCCGATTCAAGTTAGTACTAGTAG-3′) were used for PCR amplification of *cytb*. BLAST (www.ncbi.nlm.nih.gov/blast.cgi) search analysis against the amplified *cytb* fragments showed that 23 of 57 rodents from Lusaka, 24 of 48 from Namwala, and 143 of 158 from Mfuwe were *M. natalensis*.

The QIAGEN OneStep RT-PCR Kit (QIAGEN) was used to screen arenaviruses under the following conditions: 30 min at 50°C, 15 min at 95°C, 45 cycles of 20 s each at 95°C, 30 s at 50°C, 1 min at 72°C, and 10 at 72°C. The primer sequences used were 5′-CACATAGTTGGGCCCCACTTGCTGTGATC-3′ and 5′-AGGATAAGTGAAAGAGAGAGTAATTC-3′, which were designed on the basis of a consensus sequence of the large (L) gene among African arenavirus strains (Old World Arenaviruses [OWAs]), including Lujo virus. The region of the L gene has been reported as well conserved among OWAs (*7*).

Total RNA samples were extracted from kidney tissues by using TRIzol reagent (Invitrogen, Carlsbad, CA, USA). Reverse transcription (RT) PCR results indicated that 4 (17%) of the 23 rodents captured in Lusaka and 1 (4%) of the 24 captured in Namwala were positive for arenavirus, but none of the 143 rodents captured in Mfuwe were positive. Overall, 5 (3%) of the 190 *M. natalensis* rodents trapped in Zambia were positive for arenavirus. All amplicons were confirmed by nucleotide sequencing and analyzed by BLAST search.

We used RNA samples extracted from kidney tissues of representative Lusaka and Namwala strains to determine full-genome sequences. After several attempts to amplify the virus genome cDNA of OWAs by using deduced universal primers, we obtained some virus fragments and determined the full-genome sequence by the gap closing and rapid amplification of cDNA ends methods. GenBank accession numbers for the Lusaka strain are AB586644 and AB586645, and for the Namwala strain, AB586646 and AB586647. Sequence analysis indicated that the genome of the Lusaka and Namwala strains have a typical bisegmented structure containing 2 open reading frames in each segment, and the genes in the segments are separated by a stable stem-loop structure (data not shown). The small segments of the Lusaka and Namwala strains are both 3,377 bp, and the large segments are 7,230 and 7,236 bp, respectively. The shortage in the Lusaka strain genome, compared with the Namwala strain genome, was in a noncoding region between the stem-loop and the L gene.

**Figure 1:**
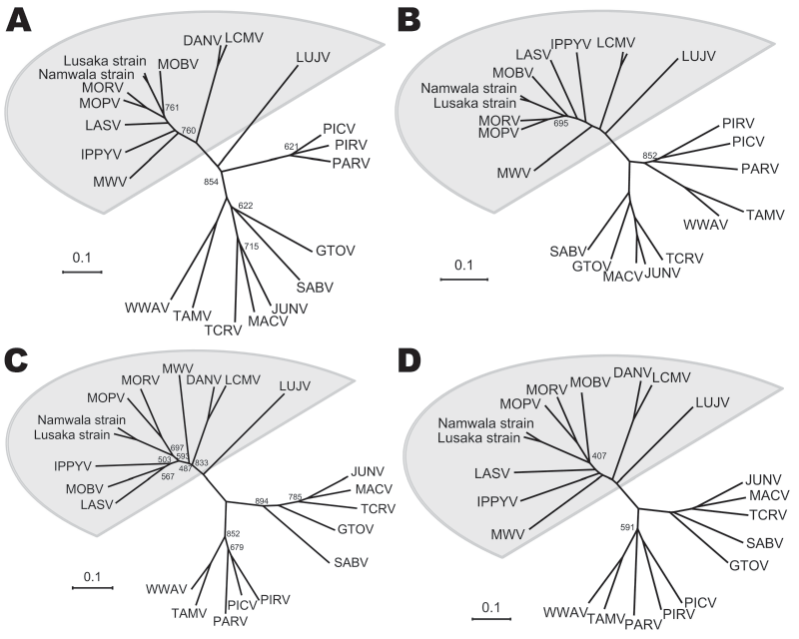
Phylogenetic analysis of Luna virus proteins based on the amino acid sequence, Zambia, 2009. Phylogenetic tree of A) glycoprotein precursor (GPC), B) nucleoprotein (NP), C) Z protein, and D) L protein. Bootstrap values are indicated in the trees (<900). Data from which amino acid sequences used for phylogenetic analyses were deduced: DANV, Dandenong virus (GenBank accession nos. EU136038 and EU136039); IPPYV, Ippy virus (GenBank accession nos. NC_007905 and NC_007906); LASV, Lassa virus (GenBank accession nos. NC_004296 and NC_004297); LUJV, Lujo virus (GenBank accession nos. FJ952384 and FJ952385); LCMV, lymphocytic choriomeningitis virus (GenBank accession nos. EU480450 and EU480453); MWV, Merino Walk virus (GenBank accession nos. GU078660 and GU078661); MOBV, Mobala virus (GenBank accession nos. NC_007903 and NC_007904); MOPV, Mopeia virus (GenBank accession nos. NC_006575 and NC_006574); MORV, Morogoro virus (GenBank accession nos. NC_013057 and NC_013058); GTOV, Guanarito virus (GenBank accession nos. AY129247 and AY358024); JUNV, Junin virus (GenBank accession nos. AY746353 and AY746354); MACV, Machupo virus (GenBank accession nos. AY624355 and AY624354); PARV, Parana virus (GenBank accession nos. AF512829 and NC_010761); PICV, Pichinde virus (GenBank accession nos.EF529746 and EF529747); PIRV, Pirital virus (GenBank accession nos. EU542420 and NC_005897); SABV, Sabia virus (GenBank accession nos. NC_006317 and NC_006313); TCRV, Tacaribe virus (GenBank accession nos. NC_004293 and NC_004292); TAMV, Tamiami virus (GenBank accession nos.NC_010701 and NC_010702); and WWAV, Whitewater Arroyo virus (GenBank accession nos. NC_010700 and [NC_0107003). Scale bars indicate amino acid substitutions per site.

We used MEGA5 software (*8*) to calculate values of diversity between the genomic small segment of the Lusaka strain and several OWAs. The lowest value, 0.13, was for Namwala strain; values ranged from 0.365 to 0.640 for the OWAs (Table 1). The divergence among each virus strain, except for the Zambian strains, ranged from 0.279 to 0.670 (Table 1). Analysis of the large segments showed similar results (data not shown). Phylogenetic analysis based on the deduced amino acid sequences of the 4 virus proteins showed clearly distinct sequences between OWAs and South American strains (New World arenavirus) (Figure 1, panels A–D; OWAs indicated in the gray area). The Lusaka and Namwala strains were classified as members of the OWAs; both strains are closely related to the Mobala, Morogoro, and Mopeia viruses. Thus, we concluded that the Zambian strains belong to the same virus species and that the novel arenavirus differs from other known strains. We propose that these Zambian strains be designated Luna virus (Lusaka-Namwala).

**Table 1:** Nucleotide sequence comparison among Old World arenaviruses*

Strain	Lusaka	Namwala	MOBV	MORV	MOPV	LASV	LUJV
Lusaka	0						
Namwala	0.130	0					
MOBV	0.365	0.367	0				
MORV	0.372	0.365	0.392	0			
MOPV	0.375	0.385	0.388	0.279	0		
LASV	0.406	0.406	0.413	0.434	0.417	0	
LUJV	0.640	0.646	0.667	0.670	0.654	0.666	0

*MOBV, Mobala virus (GenBank accession no. NC_007903); MORV, Morogoro virus (NC_013057); MOPV, Mopeia virus (NC_006575); LASV, Lassa virus (NC_004296); LUJV, Lujo virus (FJ952384). The number of nucleotide substitutions per site is shown. The small segment nucleotide sequences from 7 arenavirus strains were analyzed. All positions containing gaps and missing data were eliminated. The final dataset comprised 3,130 positions.

The characteristic functional motifs of the Z and glycoprotein precursor (GP-C) were well conserved in Luna virus. The Z protein has a critical role in arenavirus budding, and 2 conserved late-domain motifs, P(T/S)AP and PPPY, in the C-terminal have been reported (*9**,**10*). Luna virus also exhibited the P_83_TAP and P_93_PPY motifs, which are present in other OWAs, excluding Lujo, Dandenong, and lymphocytic choriomeningitis viruses (Table 2). GP-C was posttranscriptionally processed by S1P (the cellular proprotein convertase site 1 protease) to yield the glycoproteins, and the consensus motif R-(R/K/H)-L-(A/L/S/T/F) was identified as the S1P recognition site of Luna virus glycoprotein (*11**,**12*). Luna virus GP-C contained R_257_RLM, which is apparently cleaved in a similar fashion; however, its cleavage mechanism has not yet been confirmed. The RRLM sequence is also conserved in the Mobala and Ippy viruses (Table 2). The details of this protein motif suggested that Luna virus is more similar to Mobala virus than to Mopeia and Morogoro viruses.

**Table 2:** Functional motifs in the Z and glycoprotein precursor proteins of Old World arenaviruses*

Strain	Late domains†	Strain	S1P cleavage site‡
LUNV (Lusaka)	**PTAP**KESASN**PPPY**SP	LUNV (Lusaka)	RRLM-GTFTW
LUNV (Namwala)	**PTAP**KEPARN**PPPY**SP	LUNV (Namwala)	RRLM-GTFTW
MOBV	**PTAP**PPEATN**PPPY**SP	MOBV	RRLM-GTFTW
MOPV	**PTAP**PEIPPSQN**PPPY**SP	IPPYV	RRLM-STFTW
MORV	**PTAP**PEAMPSQQ**PPPY**QP	LUJV	RKLM-KLFQW
LASV	**PTAP**PTGAADSIR**PPPY**SP	MOPV	RRLL-GLFTW
IPPYV	**PSAP**PSPS**PPPY**SP	MORV	RRLL-GLFTW
LCMV	**STAP**SS**PPPY**EE§	LASV	RRLL-GTFTW
DANV	**STAP**SS**PPPY**EE	LCMV	RRLA-GTFTW
LUJV	**PSAP**PL	DANV	RRLA-GTFTW

*S1P, cellular proprotein convertase site 1 protease; LUNV, Luna virus; -, S1P cleavage site; MOBV, Mobala virus; MOPV, Mopeia virus; MORV, Morogoro virus; LASV, Lassa virus; IPPYV, Ippy virus; LCMV, lymphocytic choriomeningitis virus; DANV, Dandenong virus; LUJV, Lujo virus. †Conserved P(T/S)AP and PPPY motifs in the Z protein are in **boldface**. ‡R(R/K)LM is considered a putative, although unconfirmed, S1P cleavage site. §STAP is considered a putative, although unconfirmed, PTAP motif.

**Figure 2:**
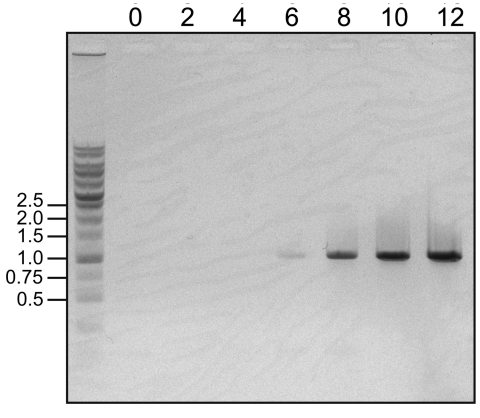
Detection of increasing viral RNA by 1-step reverse transcription PCR in a novel arenavirus, Zambia, 2009. Viral RNA was extracted from 100 μL of culture supernatant on the indicated days (top) and eluted in 20 μL of distilled water. The RNA sample was subjected to 1-step reverse transcription PCR with the specific primers 5′-TGAGAGACATTGCTTCACAATTGACATCC-3′ and 5′-TGACCCATTCTTGATGTATTGTGACTCC-3′, which were designed to amplify a 1,000-bp fragment within the determined large gene segment of Luna virus. DNA size markers are shown in the far left lane; sizes in kb are indicated at left.

We attempted to isolate Luna virus from the 5 viral RNA–positive tissue samples. Each kidney homogenate was injected into Vero E6 cells in Dulbecco modified Eagle medium supplemented with 2% fetal bovine serum. The culture medium was changed every 6 days, and the supernatant was harvested after 28 days of cultivation. The harvested culture supernatant was injected into new Vero E6 cells. During the cultivation period, culture supernatant was sampled every 2 days and tested for the presence of Luna virus RNA by 1-step RT-PCR. Finally, the amount of viral RNA in the culture supernatant of 1 Lusaka sample was increased during days 6–12 (Figure 2). During this time, distinct cytopathic effect was not observed (data not shown). To observe the virus particles, the culture supernatant was ultracentrifuged at 100,000 × *g*, and the precipitates were negatively stained with 2% phosphotungstate. Transmission electron microscopy indicated typical round-shaped, enveloped particles, 75 nm in diameter, with electron-dense dots inside the envelope (data not shown).

## Conclusions

We isolated a novel nonpathogenic arenavirus, which we propose be designated Luna virus, from *M. natalensis* rodents in Zambia. Comparison of the genetic backgrounds of Luna virus and Lujo virus, a novel pathogenic arenavirus also found in Zambia, showed that Luna virus is genetically different from Lujo virus. Luna virus was closely related to nonpathogenic arenaviruses that have been found from central to eastern Africa. To elucidate the epizootiologic aspects of arenaviruses in Zambia, the number of rodent and other species samples must be expanded. Such elucidation can lead to discovery of new arenaviruses, as demonstrated by isolation of a pathogenic New World arenavirus from bats during a study to increase knowledge of the geographic range and genetic diversity of arenaviruses naturally associated with the Mexican woodrat (*Neotoma* mexicana) in the western United States (*13*).
